# Leverage points to foster human–nature connectedness in cultural landscapes

**DOI:** 10.1007/s13280-021-01504-2

**Published:** 2021-03-08

**Authors:** Maraja Riechers, Ioana Alexandra Pătru-Dușe, Ágnes Balázsi

**Affiliations:** 1 Faculty of Sustainability, Leuphana University Lueneburg, Universitätsallee 1, 21335 Lueneburg, Germany; 2 Ecosystem Services Laboratory, Sapientia Hungarian University of Transylvania, Cluj-Napoca, Romania

**Keywords:** Human–nature relations, Land use change, Sense of agency, Sense of place, Sustainability, System change

## Abstract

Calls for a reconnection to nature and the biosphere have been growing louder over the last decades. Cultural landscapes are rapidly changing, posing a threat to ecosystems and biodiversity, but also to human–nature connections. Human–nature connectedness may be a potential lever to shift the unsustainable trajectory that we are currently proceeding, but is also negatively influenced by it. To concretize the call for a reconnection to nature, we used the leverage points perspective on five empirical case studies with focus on human–nature connectedness. Based on the synthesis of our yearlong work, in this perspective paper, we propose four leverage points to foster a sustainability transformation: (1) maintain and enhance the structural diversity of landscapes, (2) maintain and enhance economically and ecologically sustainable small-scale agriculture, (3) strengthen sense of place and (4) strengthen sense of agency in actors. Intervening in these leverage points could be effective to foster human–nature connectedness and ultimately contribute towards a sustainable trajectory. We further argue that the interconnection between leverage points is equally important as their systemic depth.

**Supplementary Information:**

The online version contains supplementary material available at 10.1007/s13280-021-01504-2.

## Introduction

Cultural landscapes are currently under change—be it through agricultural intensification and building activities or abandonment—posing threats to ecosystems (Young et al. 2005; Bürgi et al. 2017) and biodiversity (Green et al. 2005; Tscharntke et al. 2005), including the diversity of crop varieties (FAO 2011) and therewith food security (Fischer et al. 2017). Apart from ecological degradation, landscape change can negatively affect the local community structure and traditional cultural heritage of a landscape (Riechers et al. 2020a, b). We have already crossed planetary boundaries (Steffen et al. 2018) creating a sustainability gap (Fischer et al. 2007). Despite all global efforts, this sustainability gap is rather growing than closing (Fischer et al. 2007) making it necessary to focus on a deep transformation of our social–ecological systems (Meadows 1999). Yet, deep transformation has been hindered by the difficulty to tackle underlying drivers of landscape change and a focus on “easy to fix”, short-term solution (Fischer et al. 2012).

One of such transformative shift could come through the reconnection with nature (Abson et al. 2017) because it may halt the current global environmental crisis (Nisbet et al. 2009; Folke et al. 2011). While a reconnection with nature could be a remedy for an unsustainable landscape trajectory, connections to nature are also influenced by it. Recent studies have facilitated a growing recognition that landscape change erodes human–nature connectedness (HNC) (Chan et al. 2016). Many calls for ‘reconnection’ have remained vague and lack concrete insights about how to strategically foster comprehensive HNC (Ives et al. 2018) on a landscape level. In this paper, we address this research gap by presenting leverage points to foster HNC.

Leverage points are places in a complex system where small interventions can have wide ranging influences to bring about transformative system change (Meadows 1999). Leverage points have been categorized into 12 places to intervene in a system (Meadows 1999) and clustered into four system characteristics namely (1) parameters (e.g. constants, buffer stocks), (2) feedbacks (length of delay, strength of feedback), (3) system design (information flow, rules) and (4) the system intent (goals, paradigms) within which different interventions may be made (Abson et al. 2017) (see Box 1 for glossary). One of the main arguments is that these four system characteristics are encapsulated by the system with increasing order of effectiveness (from shallow to deep) for a system transformation. Therefore, interventions at deep leverage points have greater power to influence the system, while interventions targeting shallow leverage points would produce smaller changes in the system as a whole.

**Box 1:** Description of terms used regarding the leverage points perspective. These descriptions are partly direct quotes from the sources named below, partly defined or edited by the authors

● *System transformation* radical change of systemic interlinkages and systems behaviour with fundamentally different sustainability outcomes. ●* Leverage points perspective* a leverage points perspective recognizes increasingly influential leverage points from shallow to deep, encapsulated by a given system that can be used as analytical tool, metaphor and methodological boundary object. ● *Realms of leverage* overarching ‘thematic areas’ which have the influence to transform the system across all four system characteristics. *System characteristics* The four system characteristics parameters, feedback, design and intent are a nested hierarchy and tightly interlinked. Parameters and feedbacks are seen as shallow for system transformation, design and intent allow for a deeper system change. ● *Leverage points* places in a complex system where small interventions can have wide ranging influences to bring about system change and where the right kinds of intervention hold great potential for system transformation. ● *Levers* interventions that can foster change. Levers are often intuitive, but the direction in which such levers should be ‘pulled’ may not be. ● *Interventions* Concrete action taken improves situation and fosters sustainability. Levers and interventions are sometimes used interchangeably in the literature. *Sources* Meadows (1999), Abson et al. (2017), Fischer and Riechers (2019), Dorninger et al. (2020)			

We used the leverage points perspective (Fischer and Riechers 2019) as an analytical tool to illustrate four leverage points with promising potential to cause positive ripple effects across five different cultural landscapes. In this paper, we aim to understand different leverage points that foster human–nature connections, and secondly, we explore which interventions may have positive flow-on effects on the overall landscape trajectory with regard to sustainability. This perspective paper is structured as follows: First we explain the theoretical background to classify dimensions of HNC (material, experiential, cognitive, emotional, philosophical) as seen in Ives et al. (2018), and how it relates to the case studies we draw upon in the paper (Tables 1, 2). Second, we summarize the empirical background of this perspective paper (details found in supplementary S1 and S2). Third, we discuss four leverage points that may foster HNC in the five different cultural landscapes, for which we refer to the system characteristics of parameters, feedback, design and intent by Abson et al. (2017). Fourth, after this discussion, we focus on the interlinkages between those leverage points to highlight the necessity to address relationships and interdependencies of leverage points to achieve the greatest leverage potential.

**Table 1:** Dimensions of human–nature connectedness (HNC) with exemplary conceptual background and example references, and relating broad summary of the empirical results. For a more detailed description of the five dimensions of human–nature connectedness, see Ives et al. (2017, 2018). For a more detailed analysis of the different human–nature dimensions relating to the case studies, see (Balázsi et al. 2019) for a focus on the Romanian case studies, (Riechers et al. 2019) for the German case studies, and (Riechers et al. 2020a, b) for a comparison of both countries

HNC	Exemplary conceptual background	Summary of the empirical studies
Material	Focusses often on food, fuel, or other goods (Wackernagel et al. 1999; Haberl et al. 2004; Dorninger et al. 2017)	Stemming from the use of fuel (biogas, wood), food (collected, self-grown), building material, the collection of artisan goods, owning land, agriculture and forestry, and the use of regional products
Experiential	Especially activities in nature Soga and Gaston (Miller 2005; Keniger et al. 2013; Soga and Gaston 2016)	Identified as frequent nature visits, especially close to home; includes recreation, social activities in nature, stimulation of the senses, motoric development
Cognitive	Spans elements such as spirituality, aesthetics and place attachment (Kals et al. 1999; Stedman 2003; Brown and Raymond 2007)	Described as learning by doing, observing in nature through an active awareness of the daily encounters with nature, self-identification with the landscape, knowledge about the environment and farming practices, knowledge and visibility of specific historical events and cultural sites
Emotional	Captures awareness and knowledge about natural systems (e.g. Bradley et al. 1999; Schultz 2001; Schultz 2002)	Includes love for nature, spiritual and religious connections to it, aesthetics, feeling inspired and creative by being in nature, strong sense of place, curiosity to look for new and special encounters or experiences in nature, also negative emotions, such as fear and sadness regarding the state of the landscape
Philosophical	Relates to conceptions of humanity’s place in nature (e.g. van den Born 2008; Raymond et al. 2013)	Differing notions of sustainability, on consumerism and the constant need for growth, environmental protection, preservation of traditions, the highlighted responsibilities of agriculture and forestry, the definition of nature (and for whom it is)

**Table 2:** Overview of the case studies with focus on human–nature connectedness that informed the perspective piece. See also supplementary S1 and S2 *Sources* Relevant literature for Romania: (Solyom et al. 2011; Hanspach et al. 2014; Hartel et al. 2016; Horcea-Milcu et al. 2017; Balázsi et al. 2019; Klaniecki et al. 2019); for Germany: (Guerrero et al. 2012; Brandt and Glemnitz 2014; Hallmann et al. 2017; LSN 2019a; LSN 2019b)

Region	Name, county	Case study description	Methods used
Lower Saxony (Germany)	Bispingen, Lower Saxony	Partly inside the Lüneburger heath nature park. Environmental protection laws have slowed down landscape change because of restrictions to agricultural intensification and large-scale infrastructure projects. 30% of the total land area (in 2017) of Bispingen is used for agricultural practices	Qualitative interviews (*n* = 17); qualitative content analysis
	Dötlingen, Lower Saxony	More rapid landscape change over the last decades. 65% of total surface in Dötlingen is used agriculturally (in 2017), predominantly as cropland. Associated drivers have included EU agricultural subsidies and national subsidies for renewable energy production	Qualitative interviews (*n* = 17); qualitative content analysis
Transylvania (Romania)	Erdővidék, Covasna	A smallholder-dominated cultural landscape with large patches of forests, grasslands and abundant wildlife. Driven by socioeconomic and institutional change, increases in both land abandonment and intensification are considered possible in the near future	Qualitative interviews (*n* = 20); qualitative content analysis
	Aranyosszék, Cluj & Alba	Flat, crop-dominated and subject to strong urban influences due to its proximity to the cities of Cluj-Napoca and Turda. Land use intensity has increased, and smallholder vegetable cultivation has been increasingly replaced by industrial croplands	Qualitative interviews (*n* = 19); qualitative content analysis
	Pogány-havas, Harghita & Bacău	Small land holdings, with most inhabitants practising semi-subsistence farming, extensive livestock grazing, and hay meadows maintenance. The region is home to some of the most biodiverse and productive pastures and meadows in Europe and numerous threatened species	Face-to-face questionnaire (*n* = 379); statistical analysis

## Theoretical background

### Human–nature connectedness as realm of leverage

European landscapes are rich in culture as well as biodiversity with connections between humans and nature playing critical roles (Hartel et al. 2014; Elands et al. 2018). Studies show that, for example, emotional and experiential connections with nature may have positive outcomes for human well-being (Capaldi et al. 2014) or pro-environmental behaviour (Hedlund-de Witt et al. 2014), promoting in turn environmental and heritage conservation initiatives (Miller 2005). In this perspective paper, we will be using the term human–nature connectedness (HNC) to describe a myriad of connections between humans and their natural environments.

We operationalized HNC by using a multi-dimensional conceptualization (Ives et al. 2017, 2018): A material dimension, including food, fuel, and other natural goods; an experiential dimension relating to activities in nature; a cognitive dimension capturing awareness and knowledge; an emotional dimension, including spirituality, aesthetics and sense of place; and a philosophical dimension relating to conceptions of humanity’s place in nature (Table 1). We are aware that the literature of this topic is based on decades long research in various, often fragmented, disciplines, such as studies on the ‘connectedness to nature scale’ (Mayer and Frantz 2004), ‘nature relatedness’ (Nisbet et al. 2009), ‘connectivity with nature’ (Dutcher et al. 2007) or ‘relational values’ (Muraca 2011). In this paper, we do not aim to give a literature review or an in-depth analysis of those categories but give balanced and therefore simplified overarching connections of these dimensions with the leverage points perspective. For a more comprehensive overview of this topic, we therefore refer to Restall and Conrad (2015) and Ives et al. (2017).

### Methods and empirical background

In this perspective paper, we draw on five empirical case studies that have looked specifically at HNC and on our own knowledge and experience (Fazey et al. 2006) living and working in cultural landscapes. Our empirical case studies were located in Transylvania, Romania (Erdővidék, Aranyosszék, and Pogány-havas) and Lower Saxony, Germany (Bispingen and Dötlingen) (see Table 2, Figure S1). The study areas showed differing rapidity and extend of landscape changes, yet all experienced landscape simplification. Table 2 and S1/S2 show a detailed description of all five cultural landscapes and methods used.

In four study areas (Erdővidék, Aranyosszék, Bispingen, Dötlingen), we used problem-centred interviews (Flick 2006), to understand different dimensions of human–nature connectedness, the relation between these dimensions and how they are influenced by landscape change. Our interview guideline included sections on interviewees’ material, experiential, cognitive, emotional, and philosophical connectedness. Regarding landscape change, we asked specifically for perceived changes in the last 20 years, how these influenced interviewees’ lives and how interviewees perceived the trajectory of changes for the coming 20 years. We interviewed a diversity of informed laypersons and experts who we expected to be connected to a given landscape, resulting in 73 interviews (Table 1). Data were analysed using summarizing qualitative content analysis (Mayring 2008). Based on concepts used in human–nature connectedness research (Ives et al. 2017, 2018), we created a deductive coding tree which was iteratively adjusted inductively, driven by the narratives and topics raised by the interviewees.

In the Pogány-havas microregion, we used a face-to face survey (*n* = 379). The questionnaire consisted of four sections: demographics, energy acceptability, environmental values, place attachment, energy conservation attitudes and behavioural intention. Three dimensions of place attachment—place dependence, place identity and nature bonding—were assessed. We performed several analyses to understand the relationships between the dimensions of place attachment, energy conservation attitudes and behavioural intention. We further used a cluster analysis as our primary data analysis technique in an attempt to identify homogenous groups within our population that would be characterized by similar norms, practices and material culture.

Based on these empirical studies, the authors used the leverage point perspective to synthesize the separate results to this comprehensive overview. Using the original data and results, we first identified common leverage points to foster HNC in cultural landscapes and then classified these on a scale from shallow to deep.

## Results and discussion

### Leverage points to foster HNC in cultural landscapes

In the following, we highlight four concrete leverage points that can result in observable changes within a system along with their practical recommendations on how to address them. We draw from our empirical research and show examples from Romania and Germany. The leverage points are as follows: (1) maintain and enhance the structural diversity of landscapes, (2) maintain and enhance economically and ecologically sustainable small-scale agriculture, (3) strengthen sense of place and (4) strengthen sense of agency in actors.

#### Maintain and enhance the structural diversity of landscapes

Due to system-wide feedback loops (e.g. intensive land use, soil degradation), landscape complexity and ecological resilience are decreasing all over the globe (Foley et al. 2005). For example, in one of our study areas (commune Dötlingen, District Oldenburg, Germany), the percentage of area used for intensive maize production nearly doubled in 20 years (Landesamt für Statistik Niedersachsen 2018a, b) causing a homogenization of cultivated crops (e.g. Linhart and Dhungel 2013). Apart from the ecological contributions of structurally complex landscapes, our studies showed positive connections to relational values, such as cultural and individual identity (Riechers et al. 2020a), and to traditions regarding small-scale farming (Fischer et al. 2012; Molnár et al. 2015). This could mean that there is a reinforcing feedback loop between structurally complex landscapes and structurally rich social relations—that could act as a deep lever to foster sustainability (Riechers et al. 2019).

In all our five cultural landscapes, a perceived structurally complex landscape was related to several dimensions of HNC. Our study participants saw structural landscape diversity as beautiful and connecting inhabitants emotionally to landscapes. The structural landscape diversity was seen as an expression of a character of a landscape, which increased inhabitants’ sense of place. Respondents stated that places of high structural landscape diversity foster awareness and knowledge for nature and hence visited and loved them more. Yet, in all study areas, landscapes were subject to simplification through intensification of land use as well as the abandonment of agricultural land. Generally, structural landscape diversity is rooted in materials (e.g. amount of land used for certain purposes) yet influences a wide range of HNC and hence becomes a deep leverage point. For example, on a material level, structurally complex landscapes are key to protect terrestrial ecosystems and its biodiversity, especially for wild (Green et al. 2005) and farmland biodiversity, presenting a buffer for negative effects of intensive agriculture (Tscharntke et al. 2005). This means that a material connectedness may be heightened by a diverse landscape that enables a diversity of local products, while also increasing possibilities to work and relax in nature (experiential connectedness). Complex landscapes maintain diverse layers of formal and informal knowledge on nature and practices how to manage it, and can increase memories and bonds to structural elements (e.g. trees, roads, view, beauty) of the landscapes.

#### Maintain and enhance economically and ecologically sustainable small-scale agriculture

Our experiences highlight that small-scale agriculture contributed greatly to all dimensions of connectedness through, for example, regional products, sense of place, aesthetics and time spent in nature. It links to the cultural heritage of a landscape, giving it its aesthetic mosaic structure, and features of traditional management and local identity. Landscape change stemming from intensification or abandonment of agriculture can alienate inhabitants from ‘their’ landscapes emotionally, materially and experientially. Especially, small-scale farms were expressed to strengthen knowledge and interaction with nature, while large industrialized ones foster controversies about landscape ownership, economic gain and development. Yet, due to a global system intent, the land use became driven by economic efficiency, leading, in turn, to intensification and accumulation of resources in the hands of few people or companies. Farmers and foresters face common challenges all over the world (De Haan et al. 2001; Stringer et al. 2008), which is due to the prevailing global economic growth paradigm (Pedroli et al. 2007; Zimmerer 2007). A significant percentage of small-scale farms produce market commodities in Europe (Labarthe and Laurent 2013) and are of specific importance for income diversification in rural areas (European Commission 2003). Small-scale farms can alter parameters, such as those regarding biodiversity and ecological resilience (e.g. birds Nagy et al. 2009; or butterflies Konvicka et al. 2016) and can generally increase landscape complexity by enhancing crop diversities (FAO 2011).

Studies show that family farming, that is farming in shared small groups, as is traditionally practised in many areas of Transylvania has substantial production advantages to intensive farming (Mathijs and Swinnen 2001; Sabates-Wheeler 2002). These small landholdings have been managed by traditional farming practices for generations, leading to high biodiversity (Biró et al. 2011). Further, Pedroli et al. (2007) stress the economic and social benefits of small-scale farming landscapes, especially for providing identity and inspiration (see also overview of positive effects of small-scale farming in Guiomar et al. 2018). This is captured in our own empirical results (Balázsi et al. 2019) and in the literature, as inhabitants who stated to be often in nature, linked this to material goods from nature (such as food, agriculture in general, or care for own land, see also Hawkes and Acott 2013) and to cognitive connectedness through learning by doing and experience (see, e.g. Collado et al. 2013; Tekken et al. 2017). However, future changes such as farm consolidation and rural depopulation are likely to impact human–nature connections, especially in rural regions of Romania. While land use is a domain grounded within shallow leverage points as in materials (e.g. land use, production) and processes (e.g. crop rotation, fertilizer use), it is bounded by the system intent and design which limit or allow sustainable land use (e.g. agricultural policies, institutional design that implement policies).

#### Strengthen sense of place

**Fig. 1:**
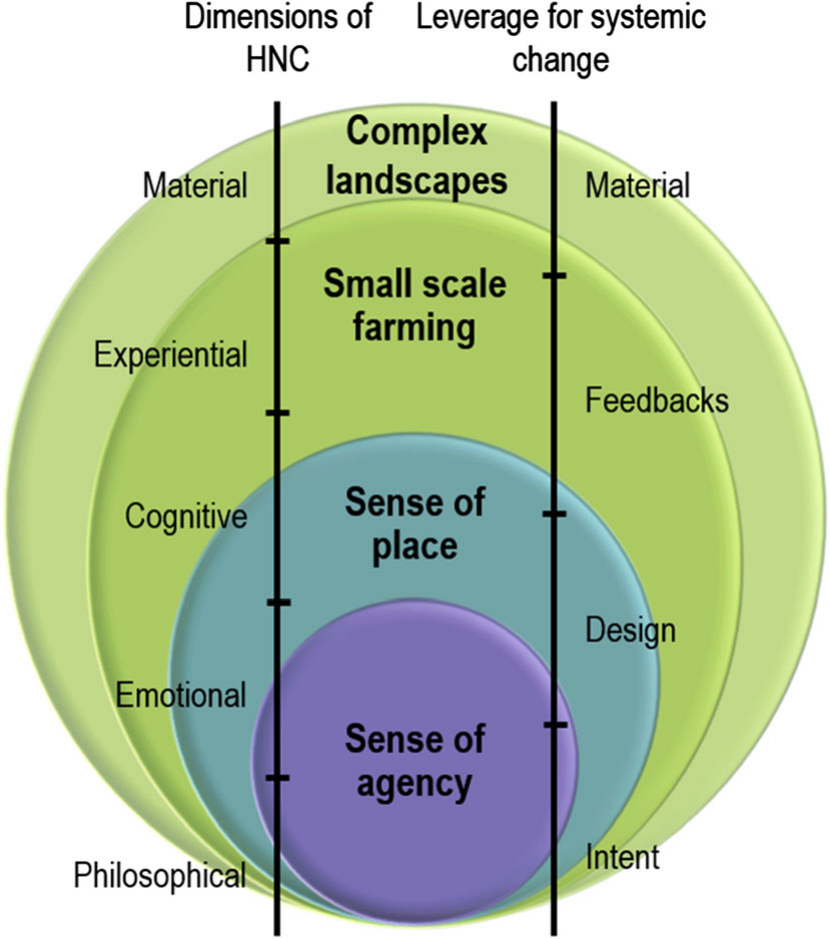
A graphical depiction of the four crosscutting themes being nested from an ecological and physical landscape attributes level (structural complexity of landscapes) to a socio-cultural level (sense of agency), showing the interdependence and relationship between the crosscutting themes. *HNC* Human–nature connectedness

Based on the definition of Williams and Stewart (1998, p. 19), sense of place is “the collection of meanings, beliefs, symbols, values and feelings that individuals and groups associate with a particular locality”. Meaning can be created through ecological (such as structural landscape diversity), social (community belonging, childhood) or social–ecological attributes (interactions with nature) by individuals or through collective meanings and shared experiences (Stedman 2002; Yung et al. 2003). Sense of place is said to combine place meanings and place attachment (Trentelman 2009; Brehm et al. 2013). In particular, place attachment is positively related to environmental action (Kals et al. 1999; Vaske and Kobrin 2001). In our case studies, a strong sense of place (emotional connection) was related to philosophical (e.g. preservation of traditions), material (e.g. regional products), cognitive (knowledge on regional history and culture) and experiential (social activities) connections to nature, bridging shallow and deep leverage points (see Fig. 1, but also e.g. Riechers et al. 2020b). Strengthening sense of place can hence increase especially emotional, experiential and cognitive connections and may empower the inhabitants and the region to gain and distribute more agency.

Our studies showed that sense of place was related not only to local identity, ethnicity, cultural identity, and languages and dialect, but also to sites of cultural heritage or natural specificity (Balázsi et al. 2019; Riechers et al. 2020b). It linked to landscape and the history of the community, to a traditional way of life including village structure or the traditional construction of buildings. Respondents perceived this strong rooted feeling of home as a possible catalyst to preserve natural areas, traditional regional products or species, and bounded the community together. In Romania, we found that residents have strong attitudes and norms towards conserving resources and that environmental behaviour is strongly rooted in being a responsible steward of natural resources and in practising frugality in the face of low incomes. We also saw in our results a potential fragility of this stewardship because of globalization-generated changes, especially when human–nature disconnections increased (Balázsi et al. 2019). Furthermore, our study participants linked the desire to maintain traditional customs to their sense of place. People in the Romanian communities highlighted the need for interventions that should focus first on developing a reliable and affordable energy supply, as this was one of their main concerns, along with supporting traditional stewardship values, conservation attitudes and practices (Klaniecki et al. 2019). Our data suggested that the loss of sense of place may have led to an alienation of inhabitants’ sense of home and belonging, including the social community to which they used to belong. Sense of place and agency seem more an expression of a systems design which can foster or hinder such expression (Riechers et al. 2019).

#### Strengthen a sense of agency in actors

The sense of agency inhabitants perceived to alter landscapes, land, and development trajectories influenced how they saw their role in nature. In parts of Germany, ownership and access to land got limited through a stronger intensification of agriculture and the accumulation of land in the hands of a few farmers. These land use changes did not necessarily correspond to inhabitants’ values of a good life, but they felt incapable of changing this trajectory of intensification. This loss of agency led to a retreat from the landscapes. Interviewees often followed up by expressing feelings of apathy or frustration, cumulating into inaction or less active involvement. This includes retreating into home gardens when, for example, surrounded by highly intensive land use, or causes emotional alienation due to strong discomfort regarding the landscape (Riechers et al. 2019). Strong feelings of agency empowered inhabitants to tackle problems with their own hands, to create knowledge exchanges or NGOs and actively, in their private and public life, tried to influence a landscapes’ trajectory to their choosing. Especially, in our study areas in Germany, we found a mismatch between inhabitants felt responsibility for landscapes and their perceived agency to alter them. This argumentation is reminiscent to social–ecological traps (Boonstra and de Boer 2014), in which behavioural responses reinforce unsustainable outcomes, here unsustainable landscape change (Steneck et al. 2011), because the system is designed in a way that restrict behavioural options. Or to use (Giddens 1984) terminology, it is comparable to structuration, a process of interactions between human action and conditions that (re)produce action. While inhabitants own desires may point towards a sustainable landscape (having their own different wish of a system goal), their own action and behaviour are guided by a system design that caters to an economic growth paradigm, inhibiting inhabitants influence on “their” landscapes and forcing farmers into ever-growing industrialized production.

Our German study sites showed that the idea of unlimited economic growth is criticized by the majority of our interviewees. Similarly, in Romania, rural inhabitants are concerned about unsustainable land use practices that occur with agricultural development, but still keep the informal knowledge of traditional farming that could be a source of inspiration for many sustainable practices. In Romania, community projects often failed because of lack of community or stakeholder support, or lack of shared information of people who have limited role in local decision-making. Additionally, limited resource availabilities and perceived sense of threat related to a further loss of place or property can make collaboration difficult. In rural communities in Transylvania, knowledge is an important driver for helping people to make informed opinions in order to become vocal and feel empowered in taking decisions for their communities. By strengthening actors’ and actor groups’ agency through, for example, informal or formal education and strengthening social cohesion (Mikulcak et al. 2015), one could create capabilities for intervention. It can also be a lever to foster self- and re-organization of a system, opening the possibility to renegotiate system goals.

### Interactions between leverage points

Based on our shared experience living and working in cultural landscapes, as well as our empirical studies, we see strong interactions between shallow and deep leverage points. Coming back to the classification of leverage points into parameters, feedback, design and intent (Abson et al. 2017), we see our four leverage points crosscutting a range of places from deep to shallow leverage points. Structural landscape diversity and small-scale agriculture are rooted in materials (e.g. land use, production) and processes (e.g. crop rotation, fertilizer use), yet both are bounded by the system intent and design which limit or allow sustainable land use. Both leverage points influence a wide range of human–nature connections and it is likely that structurally complex landscapes and structurally rich social relations may reinforce each other—acting as a deep lever to foster sustainability. Sense of place and agency seem more a combination of systems design and intent, which can strengthen or hinder such expression. Those two leverage points can also act as levers that help enable self- and re-organization of a system, opening the possibility to renegotiate its values and goals, embodied within a system of interest out of which they arise (see Fig. 1). We suggest that the interaction between shallow and deep leverage points is crucial to be understood for research and any future policy recommendations (Manlosa et al. 2018).

Interventions within these leverage points that could foster HNC are, however, scale dependent—showing differences between the individual, community or global level. In our case studies, especially the differentiation between a societal level of philosophical and material connectedness and their interplay with the individual level was relevant. A philosophical connectedness on a societal level captures much more the underlying paradigms, as exemplified by paradigms of economic wealth, social welfare and regulations and rules for environmental protection (Riechers et al. 2020b). For example, in Romania and Germany, the current paradigm is one of economic growth which is fostering telecoupling and teleconnections (Yu et al. 2013; Dorninger et al. 2017) of material flows in the regions. The societal material connectedness is hence characterized by an ever-increasing dislocation of production, use and consumption. Individuals do not have influence and agency over such globalized supply and demand chains. The possibility of this societal material connectedness to act as a leverage point is therefore limited. A shift towards a more sustainable landscape trajectory that emerges from an ecocentric worldview could be achieved by redesigning system goals on the ethics of environmental justice. This could be a powerful and deep leverage point (Schultz 2002) with multiple effects on shallow system characteristics such as parameters (e.g. environmental policies and regulations, prices of healthy products, expenditures for polluters). Further, a focus on personal sustainability of individuals could enhance a new paradigm and goal of the system (Ives et al. 2020) which might have ripple effects for the sense of agency, and sense of place people inherit (Plesa 2019; Sörqvist and Langeborg 2019).

Another linkage is the design of the system (deep leverage)—how information flows are structured, the rules of the system and the agency and power to change or self-organize the structure. In Romania, our studies point to institutional changes (through shifting political paradigms) that alienated people from the land and also created conflicts between political sectors and actors, due to unclear and conflicting legislation (van Dijk 2007; Levers et al. 2016; Balázsi 2018). One typical institutional failure is the situation of the small-scale farming (Hartel et al. 2014). Small-scale farmers, or peasants as they are preferred to be called, are marginalized and often pressured to sell or rent their lands by the agricultural industry. Further, the national food policy limits how and which products can be marketed, creating institutional barriers for small-scale farmers for additional income (Mikulcak et al. 2015). This system design fosters the growth of farmers away from small-scale agriculture and towards a more intensive, monoculture farming system (Loos et al. 2015). Similar institutional processes can be found all over the world (Mihók et al. 2015; Auer et al. 2017; Balázsi 2018). Redesigning institutions, how they function and how legislations are implemented shape the cultural landscape through its agriculture, forestry or environmental conservation and directly affect the feedback mechanism and parameters of the system.

## Conclusion

Cultural landscapes are changing and impact the way inhabitants connect to their landscapes and nature—but this connection can also impact the way landscapes will continue to change in the future. The environmental crisis of our days requires action, and our research presents possible directions to intervene in a possible spiral of disconnectedness from nature. We found four leverage points to strengthen human–nature connectedness (HNC) through our empirical studies done in five landscapes in Transylvania, Romania and Lower Saxony Germany: (1) maintaining and enhancing the structural diversity of landscapes, (2) economically and ecologically sustainable small-scale agriculture, (3) strengthen sense of place and (4) strengthen sense of agency of actors. Especially important for a sustainability transformation is the emphasis on interlinkages between these shallow and deep leverage points. All four leverage points can reinforce each other, as it is possible that that structurally complex landscapes and structurally rich social relations are linked. Redesigning the function and structure of formal and informal institutions (deep leverage point) directly affect the feedback mechanism and parameters of the system (shallow leverage point). Strengthen sense of place and agency may enable self- and re-organization of the social–ecological system by opening the possibility to renegotiate its values and goals which may ultimately enhance structural diversity of landscapes and small-scale agriculture. Our wider research showed similar examples from across the globe (Riechers et al. 2020a), making room for the hypothesis that degrading landscapes might also degrade social relations. The interaction between shallow and deep leverage points is an under researched area, and we see necessity to understand such interlinkages to foster transformative change. Further research also needs to focus on the scale dependency and agency to intervene in social–ecological systems to foster transformative change. Individual agency, for example, might be limited when tasks with refocussing a growth-centric economic paradigm which influenced land use and consumer behaviour in both countries. Yet, while our studies point to concrete leverage points, we by no means argue for a generalization of interventions and we are certain that multiple other leverage points exist that can foster HNC. Instead, with our results, we intend to highlight the importance of looking for deep leverage points that may span across multiple dimensions of HNC, highlight the interaction between shallow and deep ones and are not confined by disciplinary or geographical boundaries.

## Supplementary Information

Below is the link to the electronic supplementary material.

Supplementary material 1 (PDF 847 kb)
