# Supplementary material for: Leverage points to foster human–nature connectedness in cultural landscapes
Source: Ambio. 2021 Mar 8;50(9):1670–80. doi: 10.1007/s13280-021-01504-2 (PMC8285458; doi:10.1007/s13280-021-01504-2)
Supplement: Supplementary file 1 — Supplementary material 1 (PDF 847 kb) [file 13280_2021_1504_MOESM1_ESM.pdf]

Electronic Supplementary Material
This supplementary material has not been peer reviewed.

Title: **Leverage points to foster human–nature connectedness in cultural landscapes**

Authors: Maraja Riechers, Ioana Alexandra Pătru-Dușe, Agnés Balázsi

Supporting information S1

The social-ecological systems of Transylvania, Romania and Lower Saxony, Germany

In Transylvania, Romania we focused on the landscapes of Erdővidék (Covasna county), Pogány-havas microregion (Harghita and Bacău counties) and Aranyosszék (Cluj and Alba counties) (Figure S1). Erdővidék is a smallholder-dominated cultural landscape with large patches of forests, grasslands and abundant wildlife. Studies in biophysically comparable landscapes in Transylvania have documented high levels of wild and farmland biodiversity and a wide range of ecosystem services (Hanspach et al. 2014). Driven by socioeconomic and institutional change, increases in both land abandonment and intensification are considered possible in the foreseeable future (Hartel et al. 2016). While changes have been slow in Erdővidék to date, ongoing governance challenges and socioeconomic changes could pose a risk to the landscape and its social structures in the long run (e.g. Horcea-Milcu et al. 2017). Local industry declined in the period of socialism, and Erdővidék struggles with poor socio-economic viability and emigration of its youth (Sandu et al. 2018). Infrastructure development has increased in the last decade due to accessible EU-funds. The Pogány-havas microregion is characterized by small land holdings, with most inhabitants practicing semi-subsistence farming, extensive livestock grazing, and hay meadows maintenance (Klaniecki et al. 2019). The cultural landscape of the region is made up of large strips of forests, hay meadows, and mountainous areas with deep valleys and wide basins. The region is home to some of the most biodiverse and productive pastures and meadows in Europe (Solyom et al. 2011) and numerous threatened species (Dorresteijn et al. 2013). People in these regions have been managing their lands and rural economies for the past centuries and therefore their relationship and engagement with the area is quite special. In contrast, the landscape in Aranyosszék is flat, crop-dominated and subject to strong urban influences due to its proximity to the cities of Cluj-Napoca and Turda. Following Romania's accession to the EU in 2007, land use intensity has increased, and smallholder vegetable cultivation has been increasingly replaced by industrial croplands. Family farming is declining because of an ageing population and strong competition with supermarkets. Industrial development and small businesses have increased in Aranyosszék due to improvements in infrastructure.

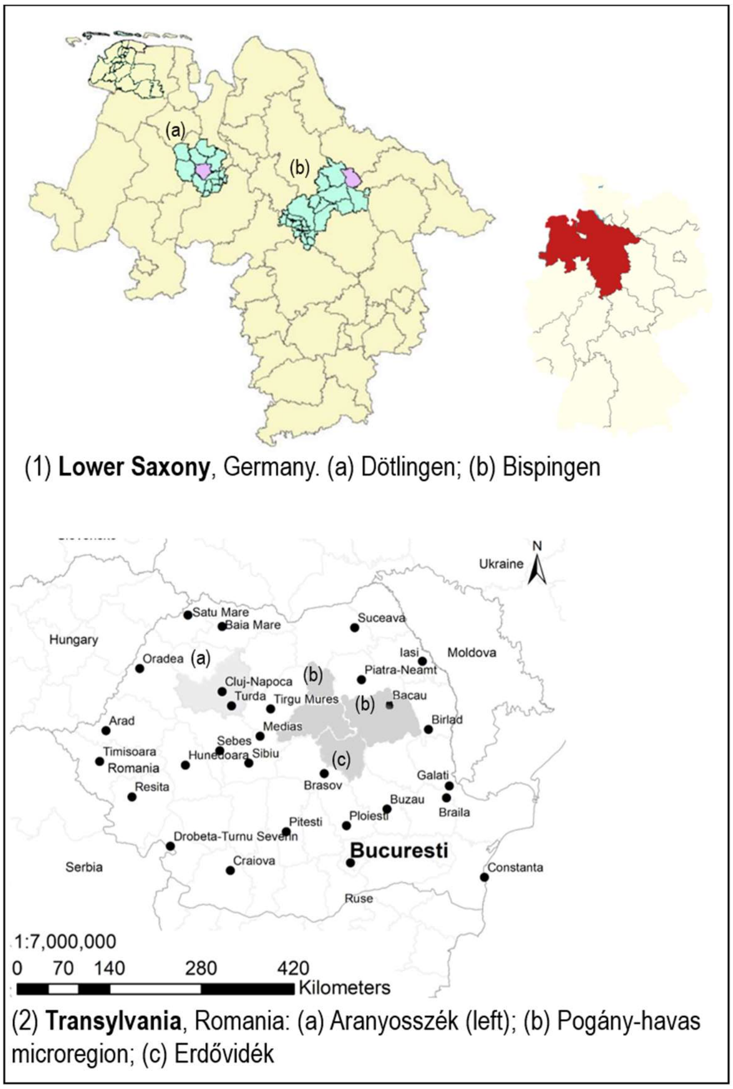

Figure S1 Location of the five study sites. (1) Lower Saxony, Germany, (a) Dötlingen (district Oldenburg); (b) Bispingen (district Heidekreis); (2) Transylvania, Romania, (a) Aranyosszék (Cluj and Alba counties); (b) Pogány-havas microregion (Harghita and Bacău counties); (c) Erdővidék (Covasna county).

In Lower Saxony, Germany we focused on Bispingen (district Heidekreis) and Dötlingen (district Oldenburg). In Lower Saxony, the agricultural landscapes have been increasingly intensified. The area used for maize cultivation in agriculturally used land reached 32% in 2016 (Landesamt für Statistik Niedersachsen 2018a; area used for silage and grain maize; Landesamt für Statistik Niedersachsen 2018b), mainly due to a change in policies fostering biogas plants. The landscape in Bispingen (district Heidekreis, 6,411 inhabitants in 2016, ~128 km² (LSN 2019b)) lies in the east of Lower Saxony and partly inside the Lueneburger heath nature park (protected under Germany's federal nature conservation act, established in

1907). Environmental protection laws have slowed down landscape change because of restrictions to agricultural intensification and large-scale infrastructure projects. 3,865ha (30% of the total land area in 2017) of Bispingen is used for agricultural practices (LSN 2019a) and tourism is important income source for people within the landscape. In the district of Oldenburg the percentage of agricultural land under maize production increased, while decreases have been observed in water and air quality (Velthof et al. 2014) and biodiversity (e.g. in birds Guerrero et al., 2012; Brandt and Glemnitz, 2014; or in insects Hallmann et al., 2017). Dötlingen covers an area of ~102 km with 6,217 inhabitants in 2016 (LSN 2019b). Sixty-five per cent of the total surface in Dötlingen (i.e. 6,628 ha) is used agriculturally (LSN 2019a), predominantly as cropland. Associated drivers have included European Union agricultural subsidies, as well as national subsidies for renewable energy production.

Supporting information S2

Data collection

In four regions (Erdővidék, Aranyosszék, Bispingen, Dötlingen) we used problem–centred interviews (Flick 2006), to understand different dimensions of human-nature connectedness, the relation between these dimensions, and how they are influenced by landscape change. Interviews were held in Romanian, Hungarian, and German using a semi-structured interview guideline, which was adjusted in parts to the interviewees' profession (Atteslander 2006). The guideline included sections on interviewees' material, experiential, cognitive, emotional and philosophical connectedness, which was assessed, among others, through questions on the use of local natural products, habits and frequency of nature visits, knowledge of nature and the landscape, perception of beauty, favorite places, and sense of place. Regarding landscape change, we asked specifically for perceived changes in the last 20 years, how these influenced interviewees' lives, and how interviewees perceived the trajectory of changes for the coming 20 years. Due to different historical events in Romania and Germany, the discussion around landscape change and its drivers led to a capturing of different time periods. While most interviewees in Romania felt a need to explain the drastic political, social and economic changes of the country starting in the socialist area from 1947 onwards, German interviewees typically focused on changes in the last 20 to 40 years. We interviewed a diversity of informed laypersons and experts who we expected to be connected to a given landscape (e.g. farmers, foresters, policy makers, long-term inhabitants, priests). The sample structure for interviews was decided through research on actors and actor groups in the study areas and through snowball sampling (Flick 2006) to gain access to important actors. This approach resulted in a total of 73 interviews of an average length of 71 minutes (Erdővidék: n = 20, Aranyosszék: n = 19, Bispingen: n = 17, Dötlingen: n = 17).

In the Pogány-havas microregion we used a face-to face survey, aiming to collect a representative sample (95% confidence, 5% margin of error): for a population size of 21,617 this would require >378 surveys distributed proportionally by commune population. A total of 379 surveys were conducted. The questionnaire consisted of four sections: demographics, energy acceptability, environmental values, place attachment, energy conservation attitudes, and behavioral intention. The items were constructed in English and translated into Hungarian and Romanian. The questionnaire was pre-tested with a small convenience sample and revised to improve unclear items and survey flow. Demographic information on gender, age, ethnicity, education, occupation, and income were collected to determine characteristics of the sample population, as well as to measure influence on place attachment. Three dimensions of place attachment—place dependence (5 items), place identity (6 items), and nature bonding (5 items)—were assessed. Environmental attitudes were measured using four items from Abrahamse and Steg (2009). Behavioral

intent was measured using one item derived from Scherbaum, Popovich and Finlinson (2008). For all items, participants responded using a five-point Likert-type scale: 1=strongly disagree, 5=strongly agree. Summated scales were constructed by combining the items belonging to each construct.

Data analysis

Interviews were transcribed verbatim and analyzed with MaxQDR Plus 12 (VERBI GmbH) and NVivo 10 (QSR international). Data was analyzed using summarizing qualitative content analysis (Mayring 2008). Based on concepts used in human-nature connectedness research (2017; Ives et al. 2018) we created a deductive coding tree which was iteratively adjusted inductively, driven by the narratives and topics raised by the interviewees. The deductive approach helped to focus existing theories and allowed comparability between the study areas; it focused primarily on the five pre-defined categories of human-nature connectedness and landscape change. The inductive approach ensured that all relevant specificities and topics not covered in the coding tree were able to be captured and unexpected statements made by interviews sufficiently covered. Codes were successively grouped together to form categories of an increasing level of abstraction.

For the quantitative survey in the Pogány-havas microregion statistical data analysis was carried out using SPSS version 25.0. We performed several analysis depending on the nature of the relationships we wanted to understand. Therefore, we tested a structural model to see the relationships between the three dimensions of place attachment, energy conservation attitudes, and behavioral intention, but we also used cluster analysis as our primary data analysis technique in an attempt to identify homogenous groups within our population that would be characterized by similar norms, practices, and material culture. Our aim was to segment the population into distinguishable Energy Cultures that could be described, compared and discussed.

To assess relationships between different dimensions of human-nature connectedness we extracted stated relationships: for example, we may have coded an interviewee's statement into the category of emotional connectedness, such as sense of place; which was related to experiential connectedness, such as social activities in nature. Another interviewee might have spoken of material connections, such as provision of food, and linked this to experiential activities, such as a high frequency of nature visits. Such relations were captured and used to illustrate relations between the abstracted categories. The same procedure was used to combine information on landscape change and categories of human-nature connectedness.

References

Abrahamse, W., and L. Steg. 2009. How do socio-demographic and psychological factors relate to households' direct and indirect energy use and savings? *Journal Of Economic Psychology* 30: 711–720. doi:10.1016/j.joep.2009.05.006.

Atteslander, P. 2006. *Methoden der empirischen Sozialforschung*. 11th ed. Berlin: Erich Schmidt Verlag.

Dorresteijn, I., T. Hartel, J. Hanspach, H. von Wehrden, and J. Fischer. 2013. The conservation value of traditional rural landscapes: the case of woodpeckers in transylvania, romania. *Plos One* 8: e65236. doi:10.1371/journal.pone.0065236.

Flick, U. 2006. *Qualitative Sozialforschung*. 4th ed. Reinbek bei Hamburg: Rowohlt Taschenbuch Verlag GmbH.

Hanspach, J., T. Hartel, A. I. Milcu, F. Mikulcak, I. Dorresteijn, J. Loos, H. von Wehrden, T. Kuemmerle, et al. 2014. A holistic approach to studying social-ecological systems and its application to southern Transylvania. *Ecology & Society* 19. doi:10.5751/ES-06915-190432.

Hartel, T., K. Olga Réti, C. Craioveanu, R. Gallé, R. Popa, A. Ioniţă, L. Demeter, L. Rákosy, et al. 2016. Rural social-ecological systems navigating institutional transitions: case study from Transylvania (Romania). *Ecosystem Health and Sustainability* 2: n/a–n/a. doi:10.1002/ehs2.1206.

Horcea-Milcu, A. I., D. J. Abson, I. Dorresteijn, J. Loos, J. Hanspach, and J. Fischer. 2017. The role of co-evolutionary development and value change debt in navigating transitioning cultural landscapes: the case of Southern Transylvania. *Journal of Environmental Planning and Management* 61: 1–18. doi:10.1080/09640568.2017.1332985.

Ives, C. D., M. Giusti, J. Fischer, D. J. Abson, K. Klaniecki, C. Dorninger, J. Laudan, S. Barthel, et al. 2017. Human–nature connection: a multidisciplinary review. *Current Opinion in Environmental Sustainability* 26-27: 106–113. doi:10.1016/j.cosust.2017.05.005.

Ives, C. D., D. J. Abson, H. von Wehrden, C. Dorninger, K. Klaniecki, and J. Fischer. 2018. Reconnecting with nature for sustainability. *Sustainability Science* 13: 1389–1397. doi:10.1007/s11625-018-0542-9.

Klaniecki, K., I. A. Duse, L. M. Lutz, J. Leventon, and D. J. Abson. 2019. Applying the energy cultures framework to understand energy systems in the context of rural sustainability transformation. *Energy policy*: 111092. doi:10.1016/j.enpol.2019.111092.

Landesamt für Statistik Niedersachsen. 2018a. Agrarstrukturerhebung, Landwirtschaftszählung.

Landesamt für Statistik Niedersachsen. 2018b. Katasterfläche nach Nutzungsarten (17) der tatsächlichen Nutzung (Gemeinde; Zeitreihe). Gebietsstand: 1.1.2015. Landwirtschaftliche Fläche (ohne Moor & Heide) von 1997, 2015. .

LSN. 2019a. Katasterfläche nach Nutzungsarten (16) der tatsächlichen Nutzung' ' (Gemeinde; Zeitreihe), Katasterfläche in Niedersachsen (Gebietsstand: 1.07.2017).

LSN. 2019b. Landesamt für Statistik,
Meine Gemeinde, meine Stadt - ausgewählte Daten auf Verwaltungseinheitsebene (VE) - Gebietss tand: 01.11.2016.

Mayring, P. 2008. *Qualitative Inhaltsanalyse. Grundlagen und Techniken*. 10th ed. Weinheim/Basel: Beltz Verlag.

Sandu, D., G. Toth, and E. Tudor. 2018. The nexus of motivation-experience in the migration process of young Romanians. *Population, space and place* 24: e2114. doi:10.1002/psp.2114.

Scherbaum, C. A., P. M. Popovich, and S. Finlinson. 2008. Exploring Individual-Level Factors Related to Employee Energy-Conservation Behaviors at Work. *Journal of applied social psychology* 38: 818–835. doi:10.1111/j.1559-1816.2007.00328.x.

Solyom, A., B. Knowles, J. Bogdan, G. Rodics, R. Biro, G. Nyíro, and A. Heron. 2011. *Small Scale Farming in the Pogany-Havas Region of Transylvania*. Pogány-havas Regional Association.

Velthof, G. L., J. P. Lesschen, J. Webb, S. Pietrzak, Z. Miatkowski, M. Pinto, J. Kros, and O. Oenema. 2014. The impact of the Nitrates Directive on nitrogen emissions from agriculture in the EU-27 during 2000-2008. *The Science of the Total Environment* 468-469: 1225–1233. doi:10.1016/j.scitotenv.2013.04.058.
